# Enhanced stroke rehabilitation outcomes through Information-Motivation-Behavioral skills model and Hospital-Community-Family ternary linkage integration: A randomized controlled trial

**DOI:** 10.1097/MD.0000000000041547

**Published:** 2025-02-28

**Authors:** Yamei Xiao, Xiaomei Xu

**Affiliations:** a Department of Neurosurgery, Affiliated Hospital of Chengdu University, Chengdu, China; b Department of Nursing Care, Affiliated Hospital of Chengdu University, Chengdu, China.

**Keywords:** clinical outcomes, hospital-community-family, information-motivation-behavioral skills model, rehabilitation, satisfaction, stroke

## Abstract

**Background:**

Stroke remains a leading cause of disability and mortality globally, necessitating effective rehabilitation strategies to improve patient outcomes. This study aimed to evaluate the effectiveness of specialized routine nursing interventions, nursing interventions based on the Information-Motivation-Behavioral Skills (IMB) model, and nursing interventions combining the IMB model with a hospital-community-family triad model in patients with acute ischemic stroke.

**Methods:**

A total of 120 patients with acute ischemic stroke were randomly assigned to one of 3 groups: Control Group (specialized routine nursing interventions, n = 40), Experimental Group 1 (IMB model-based interventions, n = 40), and Experimental Group 2 (IMB model combined with hospital-community-family triad, n = 40). Clinical outcomes, including NIHSS scores, medication adherence, and functional exercise adherence, were assessed at discharge and 6 months post-intervention. Patient satisfaction was also evaluated.

**Results:**

No differences were noted between groups at baseline. Both IMB model-based interventions (Experimental Groups 1 and 2) significantly improved NIHSS scores, medication adherence, and functional exercise adherence compared to the Control Group at both discharge and 6 months post-intervention. The combined intervention (Experimental Group 2) yielded the most pronounced improvements. Patient satisfaction was highest in Experimental Group 2, followed by Experimental Group 1 and the Control Group. These improvements were greater in Experimental Group 2 than Group 1. The adjusted multivariate regression models indicated significant improvements in all measured outcomes for the experimental groups, with lower AIC and BIC values suggesting better model fit.

**Conclusion:**

Nursing interventions based on the IMB model, particularly when combined with a hospital-community-family triad, significantly enhance clinical outcomes and patient satisfaction in stroke rehabilitation. These findings advocate for the integration of comprehensive, behavior-focused interventions in routine stroke care, addressing critical gaps in current rehabilitation practices. Future research should explore the long-term benefits and broader applicability of these interventions.

## 1. Introduction

Stroke is a leading cause of morbidity and mortality worldwide, with ischemic stroke being the most common type.^[1]^ Despite advances in acute stroke management, many survivors face significant long-term disabilities that impact their quality of life.^[2]^ Effective rehabilitation strategies are crucial to improving outcomes for these patients.^[3]^ Traditional nursing interventions primarily focus on addressing immediate clinical needs, often neglecting the comprehensive support required for optimal recovery.^[4]^

The Information-Motivation-Behavioral skills (IMB) model offers a promising framework for enhancing patient outcomes through a holistic approach.^[5,6]^ Originally developed to promote health behaviors in various contexts, the IMB model has been successfully applied to improve self-management and adherence in chronic disease populations.^[5–9]^ This model emphasizes 3 core components: providing patients with essential information about their condition, motivating them to engage in health-promoting behaviors, and equipping them with the necessary skills to manage their health effectively.^[5]^

In addition to the IMB model, integrating a hospital-community-family triad can further enhance rehabilitation by ensuring continuous support across different settings.^[10,11]^ This comprehensive approach addresses the multifaceted needs of stroke patients, fostering a supportive environment that promotes recovery and reduces the risk of recurrent strokes.^[12]^ Previous studies have examined the applicability and impact of implementing these IMB models in various clinical settings.^[8,13,14]^ However, the number of randomized trials in this regard, particularly on patients with acute ischemic stroke are scarce. In addition, they have not investigated the added benefit of integrating a hospital-community-family triad in their IMB models.

Given the potential benefits of these innovative interventions, this study aimed to evaluate the effectiveness of specialized routine nursing interventions, nursing interventions based on the IMB model, and nursing interventions combining the IMB model with a hospital-community-family triad model in patients with acute ischemic stroke. We hypothesized that IMB-based interventions, particularly when combined with the hospital-community-family triad, would result in superior clinical outcomes and higher patient satisfaction compared to routine nursing care.

## 2. Materials and methods

### 2.1. Study design and subjects

**Figure 1.**
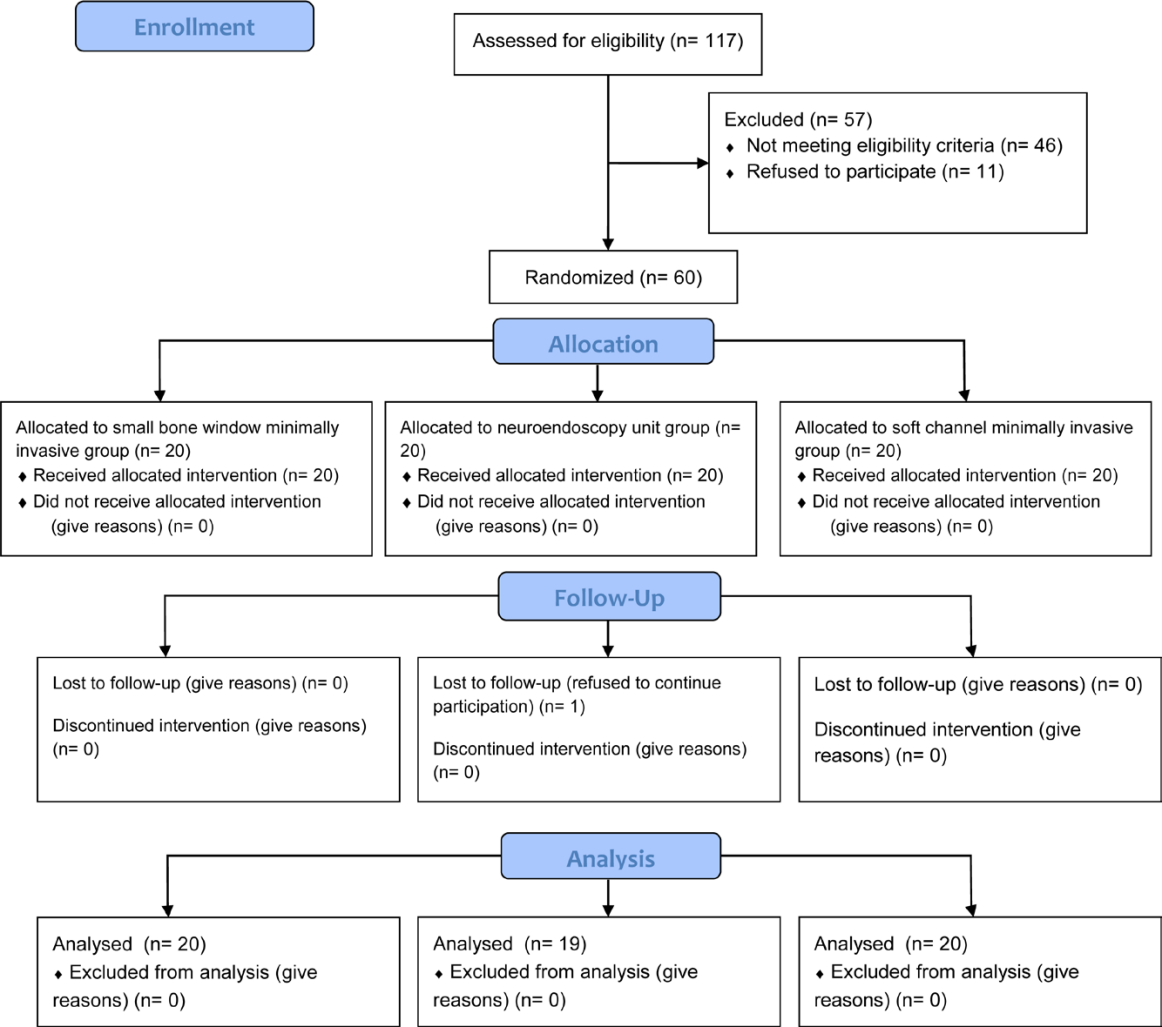
A flow chart showing the recruitment process of patients in this study.

This study included 124 patients who were hospitalized at the Neurological Medical Center (Neurology and Neurosurgery) of the Affiliated Hospital of Chengdu University and West China Hospital of Sichuan University for thrombolytic treatment of acute ischemic stroke from June 2021 to June 2023. Eligible patients were randomly assigned to one of 3 groups in a 1:1:1 ratio: Control Group (n = 40), Experimental Group 1 (n = 40), and Experimental Group 2 (n = 40) (Fig. 1). All patients underwent thrombolysis by the same neurologist. The control group received specialized routine nursing intervention, Experimental Group 1 received nursing intervention based on the IMB model in addition to the routine care, and Experimental Group 2 received a combination of specialized routine nursing, the IMB model, and a hospital-community-family triad model. The demographic and clinical characteristics of the groups were comparable (*P* > .05). This study was approved by the hospital’s ethics committee [protocol code: No:2021ZZ217, approved on 03/23/2022], and informed consent was provided by recruited patients and/or their family members/caregivers.

### 2.2. Eligibility criteria

We included patients if they met all aspects of the inclusion criteria as follows:

1. Diagnosis of acute ischemic stroke according to the criteria set by the Chinese Stroke Association.^[15]^
2. Confirmation of acute ischemic stroke via cranial CT or MRI (within 2 weeks post-stroke).
3. Age ≥ 18 years.
4. Availability of a consistent caregiver post-discharge within the Chengdu community jurisdiction.
5. First-time stroke occurrence with hospital admission within 24 hours of onset.

Meanwhile, patients were ruled out if they had any of the following criteria:

1. Severe cardiovascular, hepatic, or renal diseases.
2. Coagulation disorders.
3. Presence of tumors or mental disorders.
4. Previous lower limb surgeries affecting motor function.
5. Intervention methods

### 2.3. Randomization and blinding

Patients were randomly allocated to the 3 intervention groups using a computer-generated random number sequence. This method ensured that each patient had an equal chance of being allocated to any of the study groups, thereby minimizing selection bias. Due to the nature of the intervention, it was not feasible to blind the patients nor the research staff to the group assignments. However, the outcome assessors were blinded to the group allocations to reduce assessment bias.

### 2.4. Allocated interventions

#### 2.4.1. Control group

Patients received specialized routine nursing interventions, including:

- Pre-thrombolysis education: Nurses informed patients about the expected course of the disease to alleviate anxiety.
- Symptom monitoring: Patients were instructed to report symptoms like headache or nausea immediately for prompt treatment.
- Post-thrombolysis education: Patients were advised to remain bedridden for 24 hours post-operation and to engage in bed exercises for 3 days to prevent complications such as venous thrombosis and pressure ulcers. Post-discharge care instructions were also provided.

#### 2.4.2. Experimental group 1

This group received routine nursing care plus interventions based on the IMB model as follows:

##### 2.4.2.1. Establishment of IMB linkage team

An IMB linkage team was formed, consisting of one attending physician and one responsible nurse. The team members were trained and certified in the IMB system. Patients interacted with the team more than 5 times during their hospital stay, with each session lasting over 20 minutes.

##### 2.4.2.2. Information intervention

Nurses actively communicated with patients and their families to understand their medical history, family structure, and economic background. This communication aimed to establish trust pre-operatively and provide comprehensive information and support post-operatively, addressing common misconceptions and ensuring patients and families were well-informed about the disease and its management.

##### 2.4.2.3. Motivational intervention

Motivational interventions addressed psychological, rehabilitation, and dietary aspects:

Psychological support: Nurses provided reassurance and guidance, helping patients understand their condition and psychological stages.

Rehabilitation: Information interventions clarified the benefits of rehabilitation exercises, enhancing patient motivation and adherence to individualized rehabilitation programs.

Dietary planning: Nurses developed suitable dietary plans based on patient history, habits, and beliefs to support recovery.

##### 2.4.2.4. Behavioral intervention

Nurses assessed health behaviors during hospitalization, focusing on:

Adherence to medication and rehabilitation: Ensuring patients followed prescribed regimens and addressing any barriers.

Recovery monitoring: Communicating with patients and families to identify and address any issues in recovery, adjusting medical plans as needed.

Behavioral issues: Addressing any noncompliance due to costs or caregiver issues through early detection and communication.

#### 2.4.3. Experimental group 2

This group received a combination of routine nursing, IMB model interventions, and a hospital-community-family triad model:

##### 2.4.3.1. Establishment of triad team

The team included one attending physician, one charge nurse, one community physician, and one community nurse. All team members were trained in the IMB system. Patients communicated with the team more than 5 times during hospitalization and received bi-weekly follow-ups post-discharge.

##### 2.4.3.2. Post-discharge support

Community physicians and nurses provided continuous, personalized guidance in psychology, rehabilitation, and diet. Follow-up included answering daily nursing questions, addressing inappropriate behaviors, and actively monitoring recovery to ensure timely treatment for any serious symptoms.

### 2.5. Outcome measures

#### 2.5.1. Nursing service satisfaction

Assessed using the Newcastle Service Nursing Satisfaction Scale,^[16]^ which includes 19 questions rated from 1 (very dissatisfied) to 5 (very satisfied), with a total score ranging from 19 to 95. Higher scores indicate greater patient satisfaction with nursing services, which is linked to better compliance and improved outcomes in rehabilitation settings.

#### 2.5.2. National Institute of Health Stroke Scale (NIHSS)

The NIHSS is a comprehensive tool that assesses neurological function across 15 items, including level of consciousness, motor function, sensory loss, language, and visual fields. Scores range from 0 to 42, with higher scores indicating more severe impairment. Reductions in NIHSS scores over time are indicative of neurological improvement and are widely used to gauge recovery in stroke rehabilitation.^[17]^

#### 2.5.3. Medication adherence

Medication adherence was measured using the Medication Adherence Rating Scale (MARS), a validated 10-item questionnaire.^[18,19]^ Participants rated their adherence behaviors on a 5-point Likert scale for each item (1 = Always to 5 = Never). Total scores ranged from 10 to 50, with higher scores indicating better adherence. MARS is a widely used tool for assessing adherence in chronic conditions and was chosen for its ease of administration and relevance to the study population.

#### 2.5.4. Functional exercise adherence

Functional exercise adherence was evaluated using the scale designed by Beilei Lin et al,^[20]^ which includes 3 dimensions and 14 items, with a total possible score of 56. Higher scores indicate better adherence to functional exercises. The rehabilitation program comprised a structured set of exercises designed to address post-stroke functional recovery. Each session included range-of-motion exercises to enhance joint flexibility, strength training for muscle rebuilding, balance exercises to reduce fall risk, and aerobic exercises for cardiovascular health. Patients were scheduled for supervised sessions 3 times a week, each lasting approximately 60 minutes, with intensity and duration adapted to individual progress. Physiotherapists closely supervised the exercises to ensure proper technique and adherence. Patient engagement was monitored using attendance records and self-reported logs, while visual feedback and guidance were provided to encourage continued participation.

All outcomes were assessed at 3 specific time points: baseline (upon admission), at discharge, and 6 months post-intervention.

### 2.6. Statistical analysis

Data were analyzed using SPSS 26.0 (IBM Corp., Armonk). The Shapiro–Wilk test was used to determine normality. Normally distributed data were expressed as mean ± standard deviation (x̄ ± s) and compared using the *t* test. Categorical data were expressed as frequencies and percentages (%) and compared using the χ^2^ test. Statistical significance was set at *P* < .05.

Multivariate linear regression models were used to assess the impact of the interventions on NIHSS scores, medication adherence, and functional exercise adherence. Adjusted coefficients (β), standard errors (SE), and *P* values were reported to determine the significance and strength of associations. Model fit was evaluated using adjusted *R*^2^ values, and Akaike Information Criterion (AIC) and Bayesian Information Criterion (BIC) were used to compare model performance.^[21]^

Pearson correlation coefficients were calculated to evaluate the relationships between NIHSS scores, medication adherence, and functional exercise adherence within each intervention group. The significance of the correlations was assessed using *P* values, providing insight into how these variables interrelate within the different intervention contexts.

## 3. Results

### 3.1. Baseline characteristics

The baseline characteristics of the study participants are summarized in Table 1. One-hundred and twenty patients were initially recruited (40 patients per group); however, the data of only 115 patients were available at discharge: Control Group (n = 38), Experimental Group 1 (n = 39), and Experimental Group 2 (n = 38). The distribution of sex, age, body mass index (BMI), and duration of disease (entire period of hospitalization and access to rehabilitation resources) were compared across the groups. The chi-square test for sex distribution showed no significant difference between groups (χ²=3.789, *P* = .15). ANOVA tests revealed no significant differences in age (*F* = 1.84, *P* = .164), BMI (*F* = 2.56, *P* = .081), and duration of disease (*F* = 0.889, *P* = .414).

**Table 1:** Baseline characteristics of the 3 groups of patients

Groups	Numbers	Sex (cases)		Age (years)	Body mass index (kg/m^2^)	Duration of disease (d)
		Male	Female			
Control subjects	38	16 (42.10%)	22 (57.90%)	56.7 ± 5.3	23.1 ± 2.7	57.6 ± 8.4
Experimental group 1	39	25 (64.10%)	14 (35.90%)	54.1 ± 6.7	22.9 ± 3.3	55.9 ± 7.9
Experimental group 2	38	21 (55.26%)	17 (44.74%)	55.9 ± 6.2	24.2 ± 1.9	58.3 ± 8.1
Statistical value		χ^2^ = 3.789		*F* = 1.84	*F* = 2.56	*F* = 0.889
*P* value		*P* = .15		*P* = .164	*P* = .081	*P* = .414

### 3.2. Satisfaction scores

Table 2 presents the comparison of satisfaction scores of nursing services among the 3 groups at discharge. The satisfaction rate was highest in Experimental Group 2 (89.50%), followed by Experimental Group 1 (84.60%) and the Control Group (78.90%). The differences in satisfaction rates among the groups were statistically significant (*F* = 18.25, *P* = .02). In Experimental Group 1, a higher percentage of patients reported being very satisfied (12.82%) compared to the Control Group (5.26%).

**Table 2:** Comparison of satisfaction scores of nursing services in 3 groups

Time	Group	Cases	Very dissatisfied	Dissatisfied	Generally satisfied	Satisfied	Very satisfied	Satisfaction rate	Statistic	*P* value
At discharge	Control subjects	38	2 (5.26%)	6 (15.78%)	24 (63.15%)	4 (10.52%)	2 (5.26%)	78.90%	*F* = 18.25	*P* = .02
	Experimental group 1	39	3 (7.69%)	3 (7.69%)	13 (33.33%)	15 (38.46%)	5 (12.82%)	84.60%		
	Experimental group 2	38	2 (5.26%)	2 (5.26%)	12 (31.56%)	18 (47.36%)	4 (10.52%)	89.50%		

### 3.3. Clinical outcomes

Clinical outcomes, including NIHSS scores, medication adherence scores, and functional exercise adherence scores, were assessed at discharge and 6 months post-discharge (Table 3). At discharge, there were no significant differences in NIHSS scores (*F* = 1.414, *P* = .25), medication adherence scores (*F* = 1.149, *P* = .32), and functional exercise adherence scores (*F* = 0.728, *P* = .49) among the 3 groups.

**Table 3:** Comparison of NIHSS scores, medication adherence score, and functional exercise score in the 3 groups

Time	Group	Number of cases	NIHSS	Statistic	*P* value	Medication adherence score (MARS score)	statistic	*P* value	Functional exercise adherence score	Statistic	*P* value
At discharge	Control subjects	38	14.62 ± 3.71	*F *= 1.414	*P* = .25	43.9 ± 6.9	*F *= 1.331	*P* = .47	48.9 ± 1.6	*F *= 0.728	*P* = .49
	Experimental group 1	39	13.42 ± 2.96			45.5 ± 5.7			49.4 ± 1.5		
	Experimental group 2	38	13.59 ± 3.43			44.75 ± 8.7			49.1 ± 2.3		
6 months after Discharge	Control subjects	35	9.28 ± 1.84	*F *= 87.07	*P* < .01	31.1 ± 4.9	*F *= 88.9	*P* = .03	39.6 ± 3.6	*F *= 224.71	*P* < .01
	Experimental group 1	36	7.13 ± 1.49			37.8 ± 8.6			44.2 ± 1.8		
	Experimental group 2	36	4.69 ± 0.94			46.75 ± 8.3			51.4 ± 0.9		

MARS = Medication Adherence Rating Scale, NIHSS = National Institute of Health Stroke Score.

At 6 months post-discharge, significant improvements were observed in Experimental Groups 1 and 2 compared to the Control Group. NIHSS scores were significantly lower in Experimental Group 1 (7.13 ± 1.49) and Experimental Group 2 (4.69 ± 0.94) compared to the Control Group (9.28 ± 1.84) (*F* = 87.07, *P* < .01). The improvement in NIHSS score was also significantly greater in Experimental Group 2 than Group 1 (mean difference = *−*2.44, *P* < .01).

Medication adherence scores were higher in Experimental Group 1 (37.8 ± 8.6) and Experimental Group 2 (46.75 ± 8.3) compared to the Control Group (31.1 ± 4.9) (*P* = .03). Also, Experimental Group 2 exhibited a greater medication adherence score than Group 1 (mean difference = 8.95, *P* = .03).

Similarly, functional exercise adherence scores were higher in Experimental Group 1 (44.2 ± 1.8) and Experimental Group 2 (51.4 ± 0.9) compared to the Control Group (39.6 ± 3.6) (*F* = 224.71, *P* < .01). Better functional exercise score was noted in Experimental Group 2 than Group 1 (mean difference = 7.2, *P* < .01).

### 3.4. Multivariate analysis

Table 4 shows the adjusted multivariate linear regression models for NIHSS scores, medication adherence scores, and functional exercise adherence scores at 6 months. Compared to the Control Group, the coefficient for NIHSS scores in Experimental Group 1 was *−*2.146 (SE = 0.34, *P* = .0001, 95% CI: *−*2.821 to *−*1.471), and in Experimental Group 2 it was *−*4.56 (SE = 0.34, *P* = .0001, 95% CI: *−*5.237 to *−*3.887). The adjusted *R*^2^ values were 0.633 and 0.641, respectively, indicating substantial variance explained by the models.

**Table 4:** Adjusted multivariate linear regression models of NIHSS score, medication adherence score, and functional exercise adherence score at 6 months

		Coefficient	SE	*P*	Low CI	High CI	Adjusted R^2^	AIC	BIC
NIHSS	Experimental Group 1	−2.146	0.34	.0001	−2.821	−1.471	0.633	383.75	391.77
	Experimental Group 2	−4.56	0.34	.0001	−5.237	−3.887			
Medication Adherence (MARS score)	Experimental Group 1	3.751	0.431	.0001	2.11	4.569	0.574	399.3	413.53
	Experimental Group 2	5.983	0.473	.0001	5.103	7.059			
Functional	Experimental Group 1	4.621	0.569	.0001	3.491	5.751	0.802	493.99	502.01
	Experimental Group 2	11.732	0.569	.0001	10.602	12.862			

All analyses were adjusted for the confounding effect of age, gender, body mass index, and disease duration. All comparisons were made with the control group being set as the Reference group.

AIC = Akaike Information Criterion, BIC = Bayesian Information Criterion, CI = Confidence Interval, MARS = Medication Adherence Rating Scale, NIHSS = National Institute of Health Stroke Score, R^2^ = R-squared, a measure of model fit, SE = Standard Error.

For medication adherence scores, Experimental Group 1 had a coefficient of 3.751 (SE = 0.431, *P* = .0001, 95% CI: 2.11–4.569), while Experimental Group 2 had a coefficient of 5.983 (SE = 0.473, *P* = .0001, 95% CI: 5.103–7.059). The adjusted R² values were 0.574 for Experimental Group 1 and 0.802 for Experimental Group 2, indicating a strong model fit.

The coefficient for functional exercise adherence scores was 4.621 (SE = 0.569, *P* = .0001, 95% CI: 3.491–5.751) for Experimental Group 1, and 11.732 (SE = 0.569, *P* = .0001, 95% CI: 10.602–12.862) for Experimental Group 2. The adjusted *R*^2^ values were 0.802 for Experimental Group 1 and 0.802 for Experimental Group 2, reflecting a very good model fit. Both AIC and BIC values were lower for Experimental Group 2, indicating a better model fit.

### 3.5. Correlation analysis

Correlation analyses between NIHSS scores, medication adherence, and functional exercise adherence at 6 months, stratified by intervention groups, are presented in Table 5. In the Control Group, no significant correlations were observed between the outcomes. In Experimental Group 1, functional exercise adherence was weakly correlated with NIHSS scores (*r* = *−*0.190, *P* = .267) and moderately correlated with medication adherence (*r* = 0.299, *P* = .091). In Experimental Group 2, NIHSS scores were weakly negatively correlated with functional exercise adherence (*r* = *−*0.035, *P* = .837) and moderately positively correlated with medication adherence (*r* = 0.199, *P* = .249); however, these associations were deemed insignificant.

**Table 5:** Correlation analysis between NIHSS, medication adherence (MARS score), and functional exercise adherence scores at 6 months, stratified by the intervention groups

		Control group			Experimental group 1			Experimental group 2		
		NIHSS	Medication	Functional	NIHSS	Medication	Functional	NIHSS	Medication	Functional
NIHSS Score	Correlation	1.000			1.000			1.000		
	*P* value	–			–			–		
Medication Adherence score	Correlation	0.043	1.000		−0.307	1.000		0.199	1.000	
	*P* value	.783	–		.346	–		.249	–	
Functional Exercise Adherence Score	Correlation	−0.224	0.011	1.000	−0.190	0.299	1.000	−0.035	0.161	1.000
	*P* value	.195	.913	–	.267	.091	–	.837	.355	–

MARS = medication adherence rating scale, NIHSS = National Institute of Health Stroke Scale.

## 4. Discussion

This study aimed to evaluate the effectiveness of 3 different nursing interventions on patients with acute ischemic stroke: specialized routine nursing interventions (Control Group), nursing interventions based on the IMB model (Experimental Group 1), and nursing interventions combining the IMB model with a hospital-community-family triad model (Experimental Group 2). Given the high morbidity and mortality associated with stroke, identifying effective rehabilitation strategies is crucial for improving patient outcomes and quality of life. The decision to combine the IMB model with a hospital-community-family triad was based on the need to address multiple dimensions of support beyond the hospital setting. While the IMB model provides essential information, motivation, and behavioral skills to improve adherence and self-management, the hospital-community-family triad offers continuous support through integrated care environments. This combination aims to enhance adherence, reduce post-discharge complications, and promote long-term recovery.

Our central argument posited that incorporating the IMB model into nursing interventions, either alone or combined with a broader support network, would significantly enhance patient outcomes compared to routine nursing care. The findings of this study support this hypothesis, demonstrating that IMB-based interventions lead to better clinical outcomes and higher patient satisfaction. The study’s key findings indicate that both IMB model-based interventions significantly improved NIHSS scores, medication adherence, and functional exercise adherence at discharge and 6 months post-intervention. The improvements were more pronounced in the group receiving the combined IMB model and hospital-community-family triad intervention.

The significant reduction in NIHSS scores in the experimental groups suggests that IMB-based interventions effectively enhance neurological recovery. The IMB model facilitates this by providing patients with essential information about their condition, motivating them through personalized counseling, and equipping them with the skills necessary to manage their health proactively.^[8]^ By addressing the cognitive and behavioral aspects of recovery, the IMB model helps patients understand the importance of rehabilitation exercises and adhere to prescribed regimens, leading to better neurological outcomes.^[22]^

The higher medication adherence scores, in our study, in the experimental groups indicate that the IMB model successfully motivates patients to follow their medication regimens. This model improves medication adherence by educating patients about the critical role of medications in preventing recurrent strokes and managing stroke-related complications. Motivation is further enhanced by regular follow-ups and support from healthcare providers, reinforcing the importance of consistent medication intake.^[23]^ Additionally, the IMB model’s behavioral skills component trains patients to integrate medication routines into their daily lives effectively, overcoming barriers to adherence.^[24]^

In our study, the superior functional exercise adherence in the experimental groups underscores the importance of incorporating motivational and behavioral skills training in stroke rehabilitation programs. The IMB model helps patients understand the benefits of regular exercise for their recovery and overall health. Through motivational interviewing, patients are encouraged to set realistic and achievable exercise goals, fostering a sense of accomplishment and ongoing engagement.^[25]^ The behavioral skills component teaches patients specific exercises and how to incorporate them into their daily routines, making it easier to maintain a consistent exercise regimen.^[25]^ This adherence leads to better physical recovery and overall quality of life.

The findings of our study align with previous research on the implementation of IMB-based models in post-stroke care, as summarized in Table 6.^[8,9,13,14,26]^ For instance, Wang (2020)^[8]^ similarly reported significantly lower NIHSS scores in the IMB group with significant higher satisfaction rates, highlighting the model’s effectiveness in enhancing neurological recovery which can reflect on patients’ experience and satisfaction. Additionally, our study found higher medication adherence scores in the experimental groups, especially in Experimental Group 2. This mirrors Zheng (2024),^[9]^ who observed significant improvements in medication management with IMB model-based interventions. Meanwhile, Peng (2024)^[13]^ also demonstrated improved pulmonary function and reduced complication rates with IMB model-based care, indirectly suggesting better adherence to rehabilitation exercises. These findings reinforce the IMB model’s effectiveness in promoting regular physical activity, crucial for stroke recovery.

**Table 6:** A summary of relevant studies discussing the implementation of IMB based-models in post-stroke care

Author (YOP)	Design	Country	Population	Interventions		Follow-up	Outcome Measure	Findings
				Experimental	Control			
Wang (2020)	RCT	China	Ischemic stroke patients post-thrombolysis (n = 100)	Routine care + IMB model-based continuous nursing (n = 50)	Routine care (n = 50)	6 months	NIHSS	Significantly lower in the IMB group (*P* < .05)
							MRS	Significantly lower in the IMB group (*P* < .05)
							MMSE	Significantly higher in the IMB group (*P* < .05)
							MOCA	Significantly higher in the IMB group (*P* < .05)
							Satisfaction rate	IMB results in higher rates (*P* < .05)
Zheng (2024)	RCT	China	Ischemic and hemorrhagic stroke patients (n = 86)	Distance teaching intervention using IMB skills model plus routine care (n = 43)	Routine community health education and telephone follow-up (n = 43)	3 months	Disease management	IMB results in significant higher scores across all outcome measures at 1 and 3 months
							Medication management	
							Dietary management	
							Emotional management	
							Social functioning	
							Disease exercise management	
Peng (2024)	RCT	China	Stroke patients with pulmonary dysfunction (n = 120)	IMB model-based nursing care	Routine care	After intervention (not clarified)	Pulmonary function	IMB showed significant improvements in FEV-1, FVC, PEF compared to control
							Blood gas indices	IMB results in increased O2 saturation and O2 arterial pressure
							Complication rate	IMB group had significantly lower complications compared to control (6.67% vs. 23.33%)
							Quality of life	IMB had improved quality of life scores across all domains
Zhang (2020)	Cohort study	China	Ischemic stroke patients (n = 468)	IMB model for secondary prevention (WeChat-based)	Traditional prevention monitoring	12 months	Adherence rate	IMB results in higher odds of adherence for 8-96 weeks (OR = 2.37, *P* = .045)
							Medication noncompliance	Lower in the IMB group (3% vs. 7%, *P* = .16)
Pitthayapong (2017)	Quasi-experimental study	Thailand	Post stroke survivors (62 pairs)	Distributing pertinent information, providing skill practice during post-stroke care sessions, and utilizing strategies to enhance motivation and behavioral skills of family care givers based on IMB model (4 weeks)	Routine post-stroke care	2 months	Activities of daily living	Significantly increased over time in the IMB group (higher than control, *P* < .001)
							Complication rate	IMB group had no complications (0% vs. 6.4%, *P* = .492)

IMB = Information-Motivation-Behavioral skill model, MMSE = Mini-Mental State Examination, MOCA = Montreal Cognitive Assessment, MRS = Modified Rankin Scale, NIHSS = National Institute of Health Stroke Score, YOP = year of publication.

While our study primarily focused on clinical outcomes, the observed improvements likely contribute to better overall quality of life. This is consistent with Zhang (2020),^[26]^ who reported higher adherence rates and lower medication noncompliance in the IMB group, leading to improved long-term outcomes. Pitthayapong (2017)^[14]^ also found significant increases in activities of daily living and no complications in the IMB group, further emphasizing the model’s positive impact on daily functioning and quality of life.

Although our findings in conjunction with prior studies on the IMB model demonstrated improvements in patient adherence and satisfaction, our study adds to this by showing that integrating the hospital-community-family triad further enhances these outcomes. This finding is similar to that of Li et al,^[27]^ who found that triad-based interventions significantly impacted patients’ recovery, particularly limb function, daily living, and quality of life.

While the study’s findings strongly support the efficacy of IMB-based interventions, alternative interpretations should be considered. For example, the observed improvements could partially result from increased patient engagement due to the novelty of the intervention rather than the IMB model itself. Additionally, the combined intervention’s success might be attributed to the enhanced social support provided by the hospital-community-family triad rather than the IMB components alone.

### 4.1. Study limitations and future research directions

This study has several limitations that should be acknowledged. First, the relatively small sample size may limit the generalizability of the findings. Future studies with larger populations are necessary to confirm these results. Second, the follow-up period of 6 months may not be sufficient to capture the long-term effects of the interventions. Extended follow-up periods would provide a better understanding of the sustainability of the observed benefits. Third, the study focused primarily on quantitative outcomes and did not explore qualitative aspects of patient experiences, which could provide additional insights into the interventions’ impacts. Additionally, medication adherence, measured with the MARS scale, was based on subjective patient recall and not from pharmacy refill data, making it susceptible to social desirability bias. Lastly, the potential for selection bias exists, as patients who agreed to participate might have been more motivated or had different characteristics than those who declined.

Future research should explore these alternative interpretations by isolating the specific contributions of each intervention component. Additionally, studies with larger sample sizes and longer follow-up periods are needed to confirm the long-term benefits and sustainability of IMB-based interventions. Exploring the qualitative aspects of patient experiences could also provide deeper insights into how these interventions impact their lives and recovery journeys.

This study demonstrates that nursing interventions based on the IMB model, particularly when combined with a hospital-community-family triad, significantly improve clinical outcomes and patient satisfaction in stroke rehabilitation. These findings advocate for integrating comprehensive, behavior-focused interventions in routine stroke care, addressing critical gaps in current rehabilitation practices and offering promising directions for future research.

## Author contributions

**Conceptualization:** Xiaomei Xu, Yamei Xiao.

**Data curation:** Xiaomei Xu, Yamei Xiao.

**Formal analysis:** Yamei Xiao.

**Investigation:** Xiaomei Xu, Yamei Xiao.

**Methodology:** Xiaomei Xu, Yamei Xiao.

**Project administration:** Xiaomei Xu.

**Resources:** Xiaomei Xu.

**Software:** Xiaomei Xu.

**Supervision:** Xiaomei Xu.

**Validation:** Xiaomei Xu, Yamei Xiao.

**Writing – original draft:** Yamei Xiao.

**Writing – review & editing:** Xiaomei Xu.
